# The Role of Vertical Disparity in Distance and Depth Perception as Revealed by Different Stereo-Camera Configurations

**DOI:** 10.1177/2041669516681308

**Published:** 2016-12-06

**Authors:** Cyril Vienne, Justin Plantier, Pascaline Neveu, Anne-Emmanuelle Priot

**Affiliations:** Institut de recherche biomédicale des armées, Brétigny-sur-Orge, France; Institut de recherche biomédicale des armées, Brétigny-sur-Orge, France INSERM U1028, CNRS UMR5292, Lyon Neuroscience Research Center Bron, France

**Keywords:** vertical disparity, cue conflict, distance perception, depth perception, cameras configuration, stereoscopic displays

## Abstract

Vertical binocular disparity is a source of distance information allowing the portrayal of the layout and 3D metrics of the visual space. The role of vertical disparity in the perception of depth, size, curvature, or slant of surfaces was revealed in several previous studies using cue conflict paradigms. In this study, we varied the configuration of stereo-cameras to investigate how changes in the horizontal and vertical disparity fields, conflicting with the vergence cue, affect perceived distance and depth. In four experiments, observers judged the distance of a cylinder displayed in front of a large fronto-parallel surface. Experiment 1 revealed that the presence of a background surface decreases the uncertainty in judgments of distance, suggesting that observers use the relative horizontal disparity between the target and the background as a cue to distance. Two other experiments showed that manipulating the pattern of vertical disparity affected both distance and depth perception. When vertical disparity specified a nearer distance than vergence (convergent cameras), perceived distance and depth were underestimated as compared with the condition where vertical disparity was congruent with vergence cues (parallel cameras). When vertical disparity specified a further distance than vergence, namely an infinite distance, distance and depth were overestimated. The removal of the vertical distortion lessened the effect on perceived distance. Overall, the results suggest that the vertical disparity introduced by the specific camera configuration is mainly responsible for the effect. These findings outline the role of vertical disparity in distance and depth perception and support the use of parallel cameras for designing stereograms.

## Introduction

The vivid impression of depth in viewing stereograms is possible through the detection and scaling of binocular disparity. The detection of binocular disparity corresponds to an early stage of processing, whereby subtle differences between the images on the two retinas are measured. Disparity scaling refers to a later stage of processing, whereby cues are used to interpret horizontal binocular disparity in order to estimate depth. Specifically, disparity must be scaled using an estimate of absolute distance (Bradshaw, Glennerster, & Rogers, 1996), which is derived from various cues (Cutting & Vishton, 1995). One of these cues is the horizontal gradient of vertical disparity (Brenner, Smeets, & Landy, 2001; Rogers & Bradshaw, 1993). This pattern of vertical disparity is effective from about 20° of eccentricity, and thus, requires a sufficiently large visual field (Bradshaw et al., 1996).

The importance of vertical disparity in depth perception was revealed by a number of studies (e.g., Bradshaw et al., 1996; Brenner et al., 2001; Rogers & Bradshaw, 1993). Perceived depth, size, curvature, and slant were shown to be affected by varying the horizontal gradient of vertical disparity ( Backus, Banks, van Ee, & Crowell, 1999; Bradshaw et al., 1996; Brenner et al., 2001; Rogers & Bradshaw, 1995). For example, Brenner et al. (2001) have shown that observers do use the pattern of vertical disparity to estimate the distance of an object they are looking at. More recent empirical data also indicate that vertical disparity can affect how horizontal disparity is scaled to perceive depth when the surround (containing vertical disparity information) and the stimulus are positioned at different depth planes (O’Kane & Hibbard, 2007). It is important to note, however, that in these studies participants were not asked to judge the absolute distance. Instead, the scaling distance was derived from depth estimates. In this study, we consider the role of vertical disparity in the perception of distance and depth in stereoscopic displays as it can be revealed by improper camera configuration that provides conflicting cues to distance and depth.

Designing a stereoscopic scene requires the capture of two views, one for each eye, using two cameras set with a specific inter-axial separation. Considering real cameras, converging the axes of the cameras seems to be the correct option as the eyes also converge on the center of the region of interest. This method of converged cameras or “toed-in” is often used to make stereograms (Allison, 2007). When using real cameras and projecting on untransformed displays (i.e., on a single screen fronto-parallel to the forehead), convergent cameras can produce substantial changes in the horizontal and vertical patterns of binocular disparity.

Previous studies using geometrical analysis have shown how the geometry of a visual scene can be altered with convergent camera configuration in 3D displays (Allison, 2007; Held & Banks, 2008; Woods, Docherty, & Koch, 1993). Specifically, camera rotations introduce vertical disparities that are inconsistent with the depicted scene. These changes in vertical disparities yield a cue conflict condition between vertical disparity and other cues to absolute distance, like vergence, and therefore can affect perceived distance and depth. In addition, the use of convergent cameras affects the pattern of horizontal disparity in stereograms and thus, the overall layout of the scene (Allison, 2007). Therefore, to avoid any adverse effect of camera placement on depth perception when filming a 3D scene, it is advised that the cameras be parallel (Banks, Read, Allison, & Watt, 2012). Note however that with computer-generated imagery (CGI) or simulated cameras, the use of an asymmetric projection matrix for rendering each stereo-view avoids any unwanted transforms in the disparity field (see “*Creating* *3D* *Stereo-Views”* section).

Although convergent cameras may theoretically produce depth distortions, no experimental evidence has been provided yet (Banks, et al. 2012). On the basis of earlier analyses of the problem (Allison, 2007) and on studies of vertical disparity in depth-scaling (e.g., Brenner et al., 2001; Rogers & Bradshaw, 1993, 1995), several effects can be expected. However, it is unclear what viewers will perceive from the combination of distortions in the patterns of horizontal disparity and vertical disparity. Additionally, viewers may compensate for these effects on perceived depth as for the distortions in the case of variations in viewing position or oblique viewing (Allison & Wilcox, 2015; Hands, Smulders, & Read, 2015). Accordingly, the present study investigates how manipulating horizontal and vertical disparities can affect perceived distance and depth.

### Cue Conflict, Geometrical Analysis, and Predictions

Consider a fronto-parallel surface, located at distance *D*, the middle of which being centered on the sagittal plane. In natural vision, the eyes converge by an amount that depends on both the viewing distance *D* and the inter-ocular distance *I*. The angle of rotation of one eye relative to the line of sight while fixating a point at infinity is half the convergence angle (*c*).

**Figure 1.**
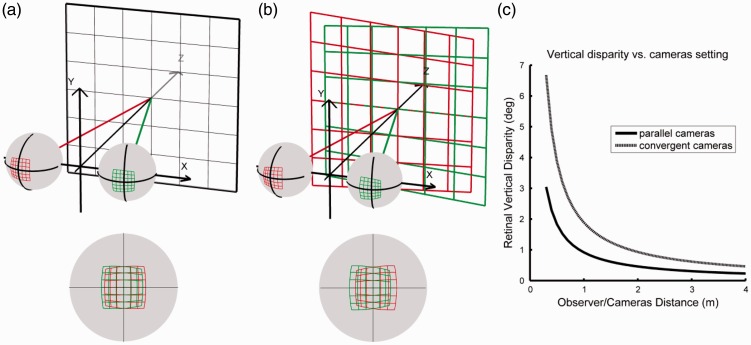
(a) Retinal projection of a fronto-parallel plane shown on both eyes (top) and the resulting horizontal or vertical disparity shown when both retinas are superimposed (bottom). (b) Retinal projection of stereograms of a fronto-parallel plane captured with convergent cameras, shown on both eyes (top), and the resulting horizontal or vertical disparity shown when both retinas are superimposed (bottom). (c) Retinal vertical disparity of a point with an eccentricity of 30°, and an elevation of 30 degrees, computed for an observer with an inter-ocular distance of 64 mm, converging symmetrically at a 1-m viewing distance. The black line is for an off-axis camera configuration, located and focusing at a 1-m distance. The dotted line represents the vertical disparity for a setting with cameras converging at 1 m, located and focusing at a 1 -m distance.

The rotation of one eye produces an oblique projection of the observed fronto-parallel plane on the retina (Figure 1(a)). The rotation of the two eyes in the opposite direction induces horizontal and vertical differences in the retinal images. The measure of the retinal vertical disparity depends on the retinal coordinate system used to describe the binocular differences (Howard & Rogers, 1995; Read, Phillipson, & Glennerster, 2009). An approximation for elevation-longitude vertical disparity is given by the following formula:

$$\mathrm{VD}=\frac{c}{2}\cdot \sin(2.e).\tan a$$

where *e* and *a* refer to elevation and azimuth as coordinates in the visual space (Read et al., 2009).

Consider now that we want to capture the same fronto-parallel surface. Converging the cameras on the center of the surface will also produce horizontal and vertical differences in the two captured images, as observed in natural vision (Figure 1(a)). As a result, the displayed fronto-parallel surface will have some obliqueness, owing to the cameras’ rotation, which is called “keystoning”. The vertical parallax (i.e., on-screen vertical disparity) from the cameras will be consistent with the cameras’ distance. However, viewers watching the stereograms of the fronto-parallel surface will also converge on the display screen. As a consequence, the vertical disparities between the left and right retinas will not match the ones usually appearing at that viewing distance (Figure 1(b)).

Figure 1(c) predicts the amount of retinal vertical disparity as a function of viewing distance and camera configuration. If an observer is placed at a viewing distance equal to that of the camera distance, and the stereo-base equals the inter-ocular distance, then the vertical disparity of the fronto-parallel surface will roughly double with convergent cameras compared with parallel ones (Figure 1(c)). Therefore, the distance specified by the pattern of vertical disparity will be halved.

If viewers judge the absolute distance exclusively from the pattern of vertical disparities, the perceived layout should be strongly distorted. Fortunately, many cues contribute to create a percept of distance (Cutting & Vishton, 1995). Perceived distance does not result solely from the pattern of vertical disparity, but rather from a compromise between the available cues (Tresilian, Mon-Williams, & Kelly, 1999). For example, in a study by Rogers and Bradshaw (1993), while the vergence angle was maintained constant, observers overestimated a depth difference (induced by a fixed 10 arcmin horizontal disparity while fixating at 57 cm) when the vertical disparity pattern specified viewing at infinity, but underestimated the same depth difference when the vertical disparity indicated viewing at 28 cm. Thus, perceived depth was influenced both by vergence and the vertical disparity pattern.

**Figure 2.**
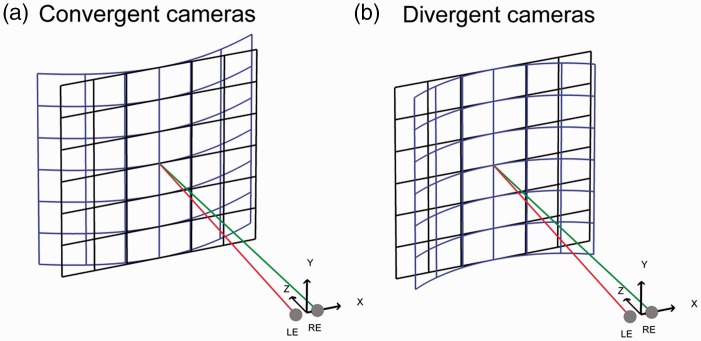
Geometrical predictions of the perceived curvature for a fronto-parallel grid (maximum of 30° eccentricity) that consider only the transforms on the pattern of horizontal disparity, for an observer with an inter-ocular distance of 64 mm sitting at 1 m. (a) Predicted percept, a convex plane (the blue grid), for displayed images taken from cameras converging on the middle of a fronto-parallel grid (in dark). (b) A concave plane, the prediction for the condition where the surface is rotated in opposite directions (divergent cameras). In both figures, LE and RE indicate the positions of the left and right eyes, whose visual axes are shown in red and green, respectively.

In addition to changing the pattern of vertical disparity, the convergent method also transforms the pattern of horizontal disparity (Allison, 2007). Considering the changes in horizontal disparity only, the layout distortions produced by a given convergent setting at a particular distance can be estimated (Figure 2(a)). As a result, the more an object is offset from the sagittal plane, the further it will be perceived away. Therefore, a fronto-parallel surface should tend to be perceived as convex, that is curved away from the observer, while the center of the object appearing at the same distance (Figure 2(a)).

Layout distortions induced by the manipulation of horizontal disparities (i.e., scene curvature) can also influence the perceived absolute distance of objects in a scene. Previous studies have shown that the relative disparity between two objects can affect the perceived absolute distance of the one closer to the observer (Blank, 1958; Foley, 1985; Sousa, Brenner, & Smeets, 2010). Glennerster, Rogers, and Bradshaw (1998) noticed that the disparities between objects with a given depth between them vary with the absolute distance. The relative disparity is larger for closer objects than for farther ones. Then small inter-objects disparities likely designate far objects distances while large disparities likely designate near objects distances. In other words, when observers have to reach a target object, the larger the relative disparity between the two objects, the more the distance of the nearer object is underestimated (Sousa et al., 2010). Thus, this effect reported for two objects should also appear in a condition with one object and a surface.

In this study, we investigate the combined effect of changes in the horizontal and vertical patterns of disparity on the perception of distance and depth. Distortions in the horizontal and vertical patterns of disparity were performed by changing the cameras configurations that were simulated using CGI virtual cameras. To study how the vertical disparity pattern can affect perceived distance, we displayed a large fronto-parallel surface located behind the stimulus target. As such, the background surface was large enough to mediate any changes in the vertical disparity field (Bradshaw et al., 1996). Additionally, the stimulus displayed in front of this surface enables the study of depth perception (i.e., the shape of the stimulus) as well as the scaling of the depth difference between the target (i.e., a cylinder) and the background surface.

## General Method

### Participants

Twelve observers took part in the entire study. They were 34.5 years old on average (*SD* = 9.1 years; range: 24–55 years). All had normal or corrected vision and presented a stereoacuity threshold less than 60 arcmin, as assessed by the TNO Test. Arm length was measured by taking the distance between the shoulder (acromion) and the tip of the index finger. All subjects provided informed consent prior to the experiment.

### Apparatus

A DLP video-projector (ACER H5360, 1280 × 720 pixels) displayed the stimuli on a projection screen (170 × 150 cm, ORAY). The participants wore 120 Hz active shutter glasses (NVIDIA 3D Vision Pro) to fuse the left and right views. The framerate was 60 Hz per view. The black level was 1.91 cd m^−2^ on the screen and was reduced to 0.02 cd m^−2^ when measured through the glasses, using a spectroradiometer (Minolta CS1000). The crosstalk percentage measured as $x=\frac{\mathrm{leakage}-\mathrm{blacklevel}}{\mathrm{signal}-\mathrm{blacklevel}}$ (Woods, 2012) was less than 0.1% in the two eyes.

To simulate distortions in the horizontal and vertical disparity fields created by a specific cameras configuration, we used virtual cameras. Stimuli were designed using OpenGL and were displayed using the PsychToolbox extension for MATLAB (Brainard, 1997; Pelli, 1997). The stereo-images were rendered as if they were obtained with specific cameras configuration: parallel or convergent. The inter-axial separation between the left and right simulated cameras was adjusted according to each individual inter-ocular distance measured with a Pupil Distance Meter (PD-82II, Towal Medical Instruments). The simulated left and right cameras were shifted by half the inter-ocular distance in opposite directions to set the cameras separation. To vary the cameras configuration, we used the convergent-axis method or the off-axis method described in the section later.

### Creating 3D Stereo-Views

Stereoscopic images can be designed by capturing left and right views spaced by a specific inter-axial separation, which usually corresponds to the inter-ocular distance of the viewer or to an average inter-ocular distance (e.g., 64 mm). There are two main options to generate stereograms using real stereoscopic cameras. A common approach to producing stereo-pairs is the convergent-axis method (or “toed-in” cameras configuration), which involves rotating the two cameras toward the direction of the target object. An advantage of this method is that it requires minimal time during postproduction (Lebreton, Raake, Barkowsky, & Le Callet, 2012). It can be easily reproduced using CGI—or virtual—cameras. By varying the degree of convergence of the cameras, one can also change the zero parallax and the range of depths (Roumes, Meehan, Plantier, & Menu, 2001). In this study, when using the convergent-axis method, the cameras symmetrically converged at screen distance. The second option uses parallel cameras and needs more postproduction routine. Both cameras are set parallel such as the visual axis is kept perpendicular to the rendering plane. Both images can be horizontally shifted to set “the plane of zero parallax”, and their edges must be cropped to adjust to the window aperture.

To test the perceptual consequences of these two types of method, it is easier to display the stimuli using CGI cameras. To simulate convergent cameras configuration, the virtual cameras must merely be rotated toward the stimulus. An alternative approach to producing stereo-pairs that simulate parallel cameras configuration is the off-axis method. To design the stereo-images, objects contained in the scene are projected onto the display plane for each camera; the position and orientation of this projection plane is the same for both camera projections. The basic principle is to set up an asymmetric frustum for each eye view and to define a near and a far clipping plane, which will include objects to-be-displayed (Jones, Lee, Holliman, & Ezra, 2001). The left and right CGI cameras are then shifted by half the inter-ocular separation of the observer from the cyclopean-eye viewpoint (located in the sagittal plane), so that the frustum is translated (see Jones et al., 2001). Figure 5 shows the resulting stereo-images for a fronto-parallel grid captured using the convergent method or the parallel one.

### Procedure and Stimuli

As stereoscopic displays present stimuli on a flat surface, they introduce a conflict between accommodation and vergence that can affect perceived distance (Vienne, Sorin, Blond窠Huynh-Thu, & Mamassian, 2014; Hoffman, Girshick, Akeley, & Banks, 2008). The size of the conflict can be characterized in terms of dioptric separation (for crossed disparity, the screen distance minus the simulated distance in diopters). Increasing the accommodation-vergence conflict can decrease depth constancy as well as fusion ability (Hoffman et al., 2008). Therefore, to display a range of portrayed object depths that was comparable across participants in terms of dioptric separation, we set the observer-display distance equal to the observer’s arm length, plus a spacing of 0.3 diopter. As a result, the observer-screen distance was 0.99 m on average. Participants judged the absolute distance (Experiments 1, 2, and 3) or the depth (Experiment 4) of vertical cylinders located at one of nine different distances (in an interval between the screen distance and a 0.5 diopter separation from that distance) presented with crossed disparity only. Stimulus depth or distance was presented using a constant stimuli method. The cylinders were displayed in front of a large fronto-parallel surface (horizontal width of 60°).

**Figure 3.**
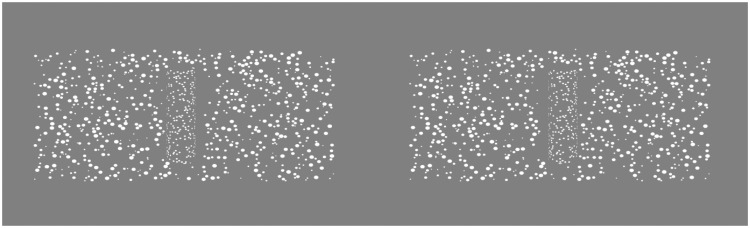
An example of a stimulus used in the study, arranged for cross fusion. The background surface and the cylinder are texture-mapped with random-dots. The task could not be performed monocularly. Dots were painted in white over a grey background to minimize crosstalk.

The stimuli were displayed using random-dot stereograms, an example of which is shown in Figure 3. The texture cue was relatively poor, due to the size of the dots. The dot size on the background surface was randomly chosen between 14 and 55 arcmin, and the dot density was 0.4 dot deg^−2^. The dot size on the cylinder was 15 arcmin, and the dot density was 0.5 dot deg^−2^.

It is important to note that changes in the perception of the apparent distance can be accompanied by changes in size perception (e.g., Gogel, 1962). As a result, to reduce the use of changing size as a cue to distance, the size of the cylinder was adjusted as a function of its intended physical distance. In other words, the angular size of the cylinder was the same across the range of displayed distances.

Participants performed the experiments in the following order: Experiment 2, Experiment 3, Experiment 1, and Experiment 4.

### Photometric Measurements

In Experiments 2, 3, and 4, dots luminance was about 134.4 cd m^−2^, and the background luminance was fixed as 25.7 cd m^−2^ (Weber contrast was 81%). In Experiment 1, dots luminance was fixed at 43.6 cd m^−2^, and the background luminance was fixed as 1.91 cd m^−2^ for Conditions 1 and 3 (Weber contrast was 96%), but dots luminance was, on average, 65.3 cd m^−2^, and background luminance was 6.6 cd m^−2^ (Weber contrast was 90%) for the Total Luminance Adjustment (TLA) condition (Experiment 1).

### Tasks

To obtain a measure of absolute distance, we asked observers to judge whether the cylinder was perceived as reachable or not. This task was used instead of a verbal report of distance because this later is known to yield a considerable variability (e.g., Pagano & Bingham, 1998). It is important to understand the relation of the perceived reachable distance to the perceived absolute distance. If a manipulation of a visual cue affects the perceived reachable distance, this is unlikely to be the result of a change in the perceived arm length or body metrics. In this condition, an overestimation of the reachable distance is more likely the consequence of a reciprocal underestimation of the absolute distance.

To obtain an estimate of perceived depth, we asked observers to indicate whether the cylinder was flattened or elongated along the depth axis. This task is analogue to the apparently circular cylinder task which requires judging the cylindricality of the stimulus (Johnston, 1991). This task requires the computation of the metric structure of the surface (Glennerster, Rogers, & Bradshaw, 1996). Some studies strongly suggest that the same estimate of egocentric distance may be used to perceive size as well as depth of objects (e.g., van Damme & Brenner, 1997). However, changes in perceived depth from depth-to-width judgments cannot be accounted by changes in perceived size only. This is because linear size (width) scales with 1/D while depth scales with 1/D^2^, D being viewing distance (Howard & Rogers, 2012). As a result, judging depth as a function of stimuli width provides a consistent measure of depth perception across various distances.

Each experiment involved three blocks of 90 trials presented in a counterbalanced order. The participant was asked to fixate a cross in the middle of the screen before the onset of each trial.

### Data Analysis

To obtain an estimate of depth or distance perception from a single measurement block, data from 90 trials were fitted with a cumulative Gaussian function with the parameters bias, α, and variance, β, using a probit regression model. To account for potential “lapses”, errors independent of stimulus features, we introduced a free parameter λ in the model: ϕ(x;α,β,λ)=(1-λ)F(x;α,β). The parameters of this function were estimated using a maximum log likelihood criterion. To obtain a measure of the perceived reachable distance, we calculated the Points of Subjective Equality (PSEs) by taking the inverse of the cumulative Gaussian function at the 50% proportion of “reachable distance”. The just-noticeable differences (JNDs, an index of uncertainty in the task) were computed by taking the difference between the point at the .75 probability level and the point at the .25 probability level and dividing this value by two. Larger JNDs indicate larger uncertainty in the task.

We performed repeated measures analysis of variance (ANOVA) after testing the assumptions of Normality and Sphericity. We ran post-hoc tests with Bonferroni correction where applicable.

## Experiment 1: Perceived Distance With a Background Planar Surface

In this experiment, the displaying method always uses asymmetric projection matrices for rendering each stereo-view. Before manipulating the vertical disparity pattern (Experiments 2 and 4), we wanted to ensure that the presence of the background surface could affect judgments of distance, as shown in some studies (e.g., Blank, 1958; Foley, 1985; Sousa et al., 2010). We investigated whether observers can use a reference surface (a fronto-parallel plane located in the screen plane) to judge the distance of a closer object located on the sagittal plane.

We thus compared the condition in which the surface was visible (“Plane” condition) with the one in which the cylinder was displayed alone (“No Plane” condition). However, removing the background surface (removing the luminous white dots) also strongly decreases the total luminance of the stereograms (i.e., the sum over the entire display), which can in turn potentially affects perceived distance and depth. For example, a reduction in luminance with contrast held constant can decrease the detection of small disparities (Richards & Foley, 1974) and increase stereo-acuity thresholds (Mueller & Lloyd, 1948). Additionally, lowering luminance is also known to alter distance perception. In low illumination, vergence and accommodation tend to return back to their resting state (tonic or dark) values, and perceived distance tends to reach a value correlated to dark vergence (Owens & Leibowitz, 1976, 1980). As a result, we also designed a condition in which the background surface was removed, and a luminance adjustment was performed (“No Plane TLA”) so that we displayed a total luminance similar to the one in the condition with the background surface.

In doing so, the gamma curve function of the projector was measured using a Konica *Minolta CS-1000*. The total luminance of the display was then adjusted according to a standard RGB model of the display response so that the total luminance in the “No Plane TLA” condition equals the one in the “Plane” condition.

To measure the change in the total luminance induced by the removal of the surface, we computed the luminance of the background surface (with and without the dots) and the one of the cylinder, knowing parameters of size and dots density. Then, the total luminance between the display of our stimuli was equated while keeping a similar luminance contrast (above 90%).

Because adding a second further stimulus in the scene helps to judge the distance of a closer one, perceived distance or uncertainty was expected to be affected.

### Results

**Figure 4.**
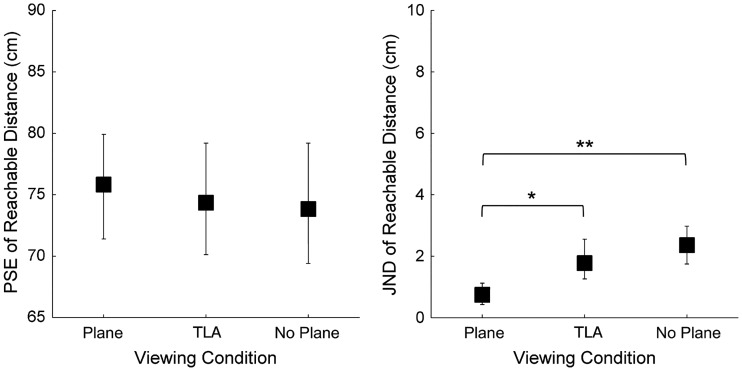
Perceived reachable distance for the three viewing conditions: stimulus in presence of a background surface (“Plane”), without the surface but with luminance adjustment (“TLA”), or presented alone without adjustment (“No Plane”). Left panel: mean points of subjective equality ; right panel: mean just-noticeable differences. Vertical error bars show 95% bootstrap confidence intervals computed using the bias-corrected and accelerated method (4,000 repetitions), Efron and Tibshirani (1994). Significant differences from post-hoc tests are indicated by * (*p* < .05) and ** (*p* < .01).

Task performance was assessed by computing distance error (estimation of reachable distance minus actual reachable distance). On average, observers overestimated the reachable distance by about 2 cm (*SD* = 7.3 cm). Figure 4 shows the results on perceived reachable distance of Experiment 1. The analysis revealed no effect of the viewing condition on PSEs, *F*(2,22) = 0.99, *p* = .39. However, the viewing condition significantly affected the uncertainty in the task, *F*(2,22) = 10.62, *p* < .001. JNDs increased with the removal of the background surface (Plane vs. TLA, *p* < .02; Plane vs. No Plane, *p* < .01), but there was no effect of the luminance adjustment (*p* > .05).

**Figure 5.**
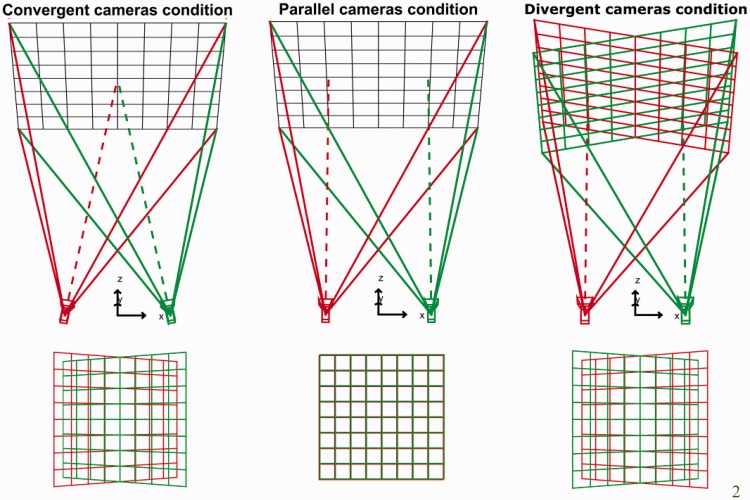
The design of stimuli in Experiment 2. Left: Cameras are set convergent leading to distortions in the horizontal and vertical patterns of disparity. Each camera frustum is symmetric. The cameras axis is directed toward the center of interest. The distortions occurred because the rendering plane and the viewing plane have different orientations. Middle: Cameras are set parallel. In the off-axis method, an asymmetric frustum is defined and allows that the rendering plane and the viewing plane match and have the same orientation. No distortions are present; the fronto-parallel plane remains the same. Right: Cameras are also set parallel. However, the images are now rotated, keeping their center aligned with each together, so as they are perpendicular to the line of sight of each eye (divergent cameras).

### Discussion

On average, observers overestimated their action space by about 4.2% (*SD* = 0.08), which is consistent with literature data (Carello, Grosofsky, Reichel, Solomon, & Turvey, 1989; Rochat & Wraga, 1997). The display of the background surface did not change the perceived absolute distance of the cylinder (PSEs). We observed a large inter-individual variability. Some observers perceived the cylinder nearer, and others had difficulty seeing the object go out of the screen plane. The pool of participants was too small to identify these two trends precisely. However, it is conceivable that the mean perceived distance was not affected because subjects did not rely on the same cues to estimate distance. For example, some participants might assign less weight to the vergence cue in favor of others (e.g., the accommodation or focus cue which signals screen distance, Vienne, Blondé, & Mamassian, 2015), and thus, they perceive less depth between the object and the screen.

However, the removal of the background surface did induce more variability in judging the cylinder distance, as revealed by increased JNDs. The increased uncertainty could be a result of the removal of the relative disparity between the object and the surface.

Sousa et al. (2010) found that reaching movements to a near object are more accurate when a distant object is added. The different results between Sousa et al.’s (2010) study and the present work are probably due to methodological differences. While requiring distance estimates, pointing to a target (Sousa et al., 2010) and judging reachability are motor and perceptual tasks, respectively. Reachability judgments are not made under the same constraints as reaching. The relative disparity between two objects limits possible distances for the nearer object (see Sousa et al., 2010 for the limiting-factor hypothesis) and potentially helps observers to judge distance. Thus, adding a more distant object may reduce variable errors (as observed in the present study) and absolute errors in motor and perceptual tasks (as observed in Sousa et al., 2010).

Finally, we did not observe any effect of the TLA condition on perceived distance between the two conditions where the background surface was removed. Presumably, the change in luminance induced by removing the background surface was not sufficient to affect either distance or depth perception.

In conclusion, observers can use the information provided by a background surface to judge the distance of a closer object. Thus, varying the information specifying the distance of the surface, by manipulating vertical parallax from cameras configuration, could also affect the perceived distance of a closer object.

## Experiment 2: The Effect of Convergent Cameras on Distance Perception

In Experiment 2, we investigated how perceived distance of simulated stereoscopic content is affected by the configuration of the stereo-cameras. Using OpenGL, we displayed stimuli using either convergent or parallel cameras (Jones et al., 2001). Parallel cameras do not introduce vertical parallax, and therefore, the distance specified by the pattern of vertical disparity is the same as the intended distance. Convergent cameras introduce vertical parallax so that the vertical disparities convey a smaller distance than the distance specified by other cues, such as vergence (Held & Banks, 2008). To further investigate this cue conflict, we also tested a third condition—a cue conflict condition—in which the vertical disparity of the background surface specified a farther distance, that is an infinite distance (Figure 5), whereas vergence specifies a closer distance. To generate the vertical disparity that would be created by a surface located at an infinite distance, we rotated the background plane such that the surface was perpendicular to the line of sight in each eye (Rogers & Bradshaw, 1995).

Observers rely on a trade-off between the available distance cues to provide their distance estimates (Hillis, Watt, Landy, & Banks, 2004; Landy, Maloney, Johnston, & Young, 1995). In the convergent-axis method, vertical disparity indicates a closer distance than in the parallel method; as a result, observers should tend to underestimate the distance of the cylinder. In the task used in the present experiment, we predicted that observers would overestimate the reachable distance. If, in the third condition, the vertical disparity specifies an infinite distance, observers should overestimate the egocentric distance and underestimate the reachable distance.

### Results

**Figure 6.**
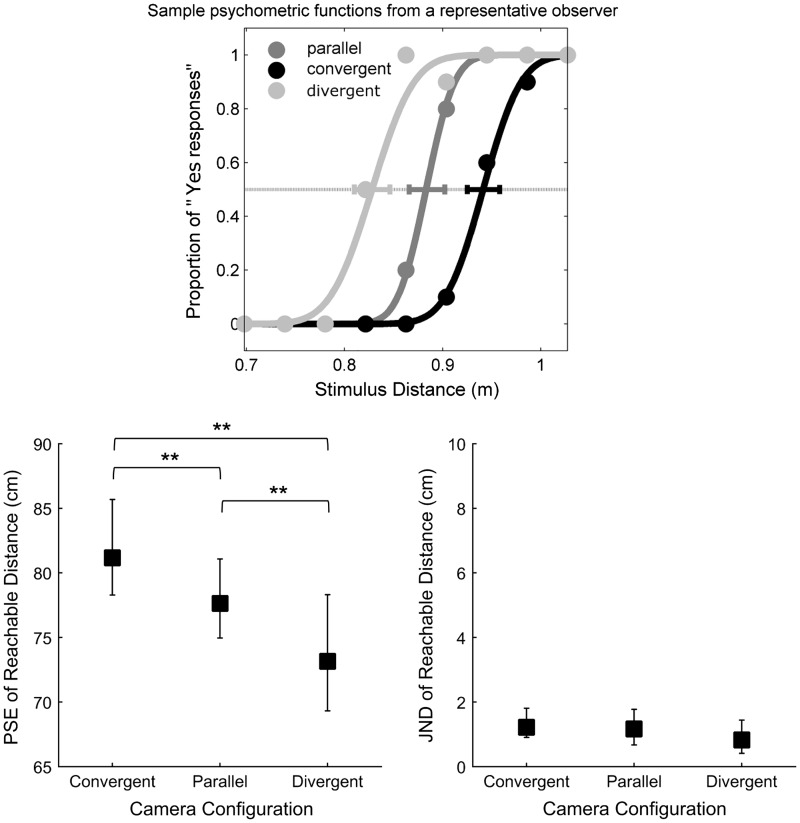
Top: Sample psychometric functions for one representative observer. Horizontal error bars show bootstrap confidence intervals of points of subjective equality (estimated using bootstrap resampling). Bottom: Perceived reachable distance for the three camera conditions: “Convergent,” “Parallel,” and “Divergent.” Left panel: mean points of subjective equality; right panel: mean just-noticeable differences. Vertical error bars show 95% bootstrap confidence intervals computed using the bias-corrected and accelerated method (4,000 repetitions), Efron and Tibshirani (1994). Significant differences from post-hoc tests are marked with a ** (*p* < .01).

Figure 6 shows the mean PSEs and JNDs for this experiment. ANOVAs showed a statistically significant effect of the camera configuration for PSEs, *F*(2,22) = 11.66, *p* < .0001. With convergent cameras, observers overestimated the reachable distance, compared with parallel cameras (*p* < .01). In the infinite-distance condition, observers underestimated the reachable distance, compared with parallel cameras (*p* < .01). However, no effect of camera configuration on JNDs was found, *F*(2,22) = 1.031, *p* > .05.

### Discussion

The results of this experiment indicate that judgments of reachable distance in stereograms can be influenced by the setting of stereo-cameras set during image acquisition. With convergent cameras, observers overestimated the reachable distance, compared with parallel cameras; the cylinder was perceived closer because of a compressed visual space. In the infinite-distance condition, observers underestimated the reachable distance, compared with parallel cameras. The cylinder was perceived farther away, presumably, because the perceptual visual space was expanded.

As discussed in the “Introduction” section, filming with convergent cameras can create a cue conflict between the horizontal gradient of vertical disparity and other cues to distance, like vergence and accommodation. Among several possibilities (for a review, see Howard & Rogers, 1995), weighted linear models have been used to describe cue combination in distance perception (Tresilian & Mon-Williams, 1999). In these models, the weight assigned to a cue is related to its reliability which is proportional to the inverse of its variance (Landy et al., 1995; Massaro, 1988). As the visual system combines information from the available cues, a compromise between these cues is required when they are conflicting.

Convergent cameras and the divergent cameras condition produced a distortion in the pattern of horizontal disparities (Figure 2(a) and (b)), in addition to the vertical distortion (Rogers & Bradshaw, 1995). It is unclear whether the effects reported in Experiment 2 resulted from distortions in the pattern of vertical disparities (Figure 1) or from distortions in the pattern of horizontal disparities (Figure 2(a)). To address this issue, we performed an additional experiment where the distortion in the pattern of vertical disparities (i.e., the vertical parallax) was removed.

## Experiment 3: The Curvature Effect Induced by Horizontal Disparities

This experiment sought to determine whether distortions in the pattern of horizontal disparity (the curvature effect) can affect distance perception. The same conditions as in Experiment 2 were used; however, the vertical parallax resulting from the camera configuration was removed. To cancel the vertical distortion, we computed the resulting vertical parallax and presented trapezoidal surfaces. Hereafter, the convergent camera condition, the parallel condition, and the divergent cameras condition are referred to as the convex-distortion, flat (or no-distortion), and concave-distortion conditions, respectively. These were the three layout-distortion conditions. As in the condition where the cameras were parallel, all conditions of the present experiment did not display any vertical parallax on the screen.

As mentioned earlier, adding a distant object helps judge the distance of a nearer one (Blank, 1958; Foley, 1985; Sousa et al., 2010). Sousa et al. (2010) found that the perceived distance of the nearer object is more and more underestimated as the relative uncrossed disparity between the two objects increased. Large inter-object disparities are usually associated with closer absolute distance, and small relative disparities are associated with farther distance (Glennerster et al., 1998). As a consequence, if observers use the relative disparity between two objects, the distortion in the pattern of horizontal disparity could affect perceived distance for a near object and a background surface in the same way than Sousa’s findings for two distinct objects. Convergent cameras modify the pattern of horizontal disparity such as the horizontal disparity in the background surface increases with eccentricity (a convex surface, Figure 2(a)). Observers could also perceive a fronto-parallel surface as being on average farther away, rather than perceiving a convex surface. As a result, if the horizontal disparity field is sampled over the entire surface, this would lead observers to perceive the nearer object as being closer than it actually is. The divergent cameras condition affects the horizontal disparity pattern so that the horizontal disparity in the surface decreases with eccentricity (a concave surface, Figure 2(b)). Observers could also perceive the fronto-parallel surface as being on average nearer, rather than perceiving a concave surface. Again, this would lead observers to perceive the nearer object as farther away. These predictions have an analogous directional effect to those drawn from the effect of vertical disparity (Experiment 2).

### Results

**Figure 7.**
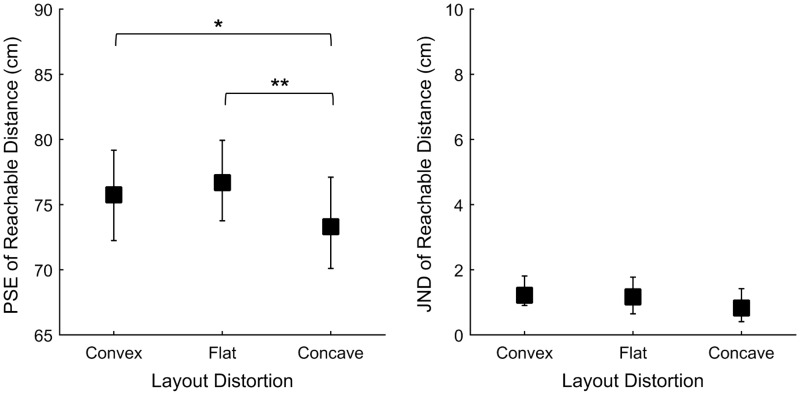
Perceived reachable distance for the three viewing conditions: convex-distortion, “Flat” or no-distortion, and concave-distortion, where vertical parallax was removed. Left panel: mean points of subjective equality; right panel: mean just-noticeable differences. Vertical error bars show 95% bootstrap confidence intervals computed using the bias-corrected and accelerated method (4,000 repetitions), Efron and Tibshirani (1994). Significant differences from post-hoc tests are marked with * (*p* < .05), ** (*p* < .01).

Figure 7 shows the results of Experiment 3. ANOVAs showed a statistically significant effect of the layout-distortion condition on PSEs, *F*(2,22) = 13.53, *p* < .0001. However, the convex-distortion (convergent cameras) did not significantly affect the reachable distance when compared with parallel cameras (*p* > .05). In the concave-distortion condition, observers underestimated the reachable distance compared with both the no-distortion condition (*p* < .01) and the convex-distortion condition (*p* < .05). We found no effect of the distortion in the horizontal pattern of disparity on JNDs, *F*(2,22) = 0.98, *p* > .05.

### Discussion

We predicted that distortions in the pattern of horizontal disparities would lead to underestimate the perceived distance of the nearer object when using convergent cameras. Comparing the results of the convex-distortion (convergent cameras) and no-distortion (parallel cameras) conditions, we found that distortions in the pattern of horizontal disparity had no significant effect on distance perception. This suggests that the effect of convergent cameras, observed in Experiment 2, mainly occurred because of distortions in vertical disparities.

However, we observed a significant difference between the results of the concave-distortion condition (divergent cameras condition) and of the two other conditions. Thus, a role of horizontal disparities cannot be ruled out in this condition or in Experiment 2. As proposed in the “Introduction” section, this result likely suggests that the relative disparity between the stimulus and the background surface influences perceived distance, consistent with the effect observed between two objects by Sousa et al. (2010) and Sousa, Brenner, and Smeets (2011).

## Experiment 4: The Effect of Convergent Cameras on Depth Perception

Manipulating the pattern of vertical disparity can affect distance perception in stereoscopic content. The perceptual estimation of absolute distance affects how horizontal disparities are scaled to estimate depth (Bradshaw et al., 1996). Therefore, we investigated the effect of this manipulation on depth perception.

Because the horizontal disparity between a pair of points varies inversely with the square of the absolute distance (Kaufman, 1974), perceiving depth from horizontal disparity requires scaling disparity with absolute distance information. Thus, any change in the available cues to absolute distance should affect perceived depth. For example, the horizontal disparity yielding an object’s depth normally becomes greater as the absolute distance decreases. If observers underestimate the apparent distance, the horizontal disparity between a pair of points should also be underestimated as it is actually smaller than the disparity of a pair of points at that perceived distance. As a consequence, the underestimation of the absolute distance (as in the convergent camera condition) should lead observers to judge the cylinder depth as being smaller. If the absolute distance is overestimated (as in the divergent cameras condition), observers should perceive the cylinder depth as larger. Finally, because the vertical disparity pattern influences perceived distance and because distance information is used to scale disparity, perceived distance and depth might be influenced in the same way.

To investigate how perceived depth is influenced by the vertical disparity setting, observers were required to estimate the depth of a cylinder (i.e., to indicate which, of the depth or the width of the cylinder, was larger), which was fixed at reachable distance. To help in the task, we displayed a transparent cylinder, so that observers could use the front and back face of the cylinder to better estimate its depth (Vienne et al., 2015). In this experiment, we used the method of constant stimuli to vary the depth ratio of the cylinder (i.e., the cylindricality).

### Results

**Figure 8.**
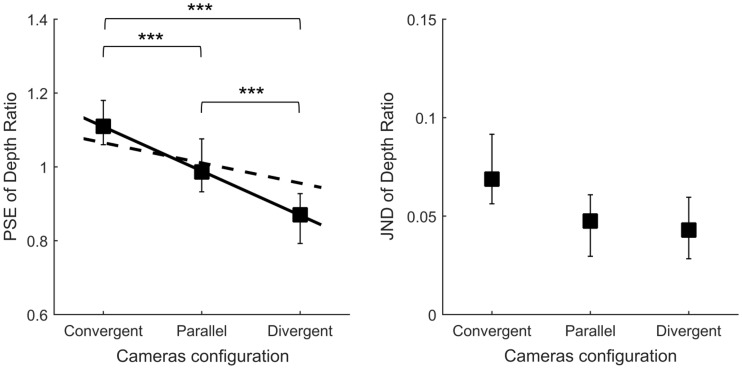
Depth ratios providing the perception of a perfectly circular cylinder for the three experimental conditions: “convergent,” “parallel,” and “divergent.” Depth ratios above 1 correspond to underestimation of depth, and depth ratios below 1 show depth overestimation. Left panel: mean points of subjective equality. The dotted line represents the predicted depth ratios based on the perceived distance measured in Experiment 2. The solid line is the regression line performed on the measured depth ratios. Right panel: mean just-noticeable differences. Vertical error bars show 95% bootstrap confidence intervals computed using the bias-corrected and accelerated method (4,000 repetitions), Efron and Tibshirani (1994). Significant differences from post-hoc tests are marked with *** (*p* < .001).

Figure 8 shows the results obtained from Experiment 4 and predictions drawn from Experiment 2. Depth ratios are the scaled depth over displayed depth required to perceive a perfectly circular cylinder. As a consequence, depth ratios above 1 indicate depth underestimation whereas depth ratios below 1 indicate depth overestimation. ANOVAs revealed an effect of the cameras configuration on the PSEs of depth ratios, *F*(2,22) = 41.38, *p* < .0001. Convergent cameras led observers to underestimate the depth of the cylinder compared with parallel cameras (*p* < .001). In the divergent cameras condition, observers overestimated the cylinder depth compared with the parallel cameras condition (*p* < .001). However, we found no effect of the camera configuration on JNDs, *F*(2,22) = 2.56, *p* > .05.

Additionally, the figure shows the predicted depth computed from individual data obtained on distance estimation in Experiment 2. We performed an additional statistical analysis to compare the depth ratios predicted from the perceived distance and the measured depth ratios. Linear regressions were performed on the individual data to characterize the effect of the horizontal pattern of vertical disparity. A *t* test revealed that the unsigned slopes of the measured depth ratios were significantly larger than those of the predicted ones, *t*(11) = 5.34, *p* < .001, therefore indicating a greater effect for perceived depth than the one we could expect from perceived distance.

### Correlation Analysis: Distance Versus Depth

**Figure 9.**
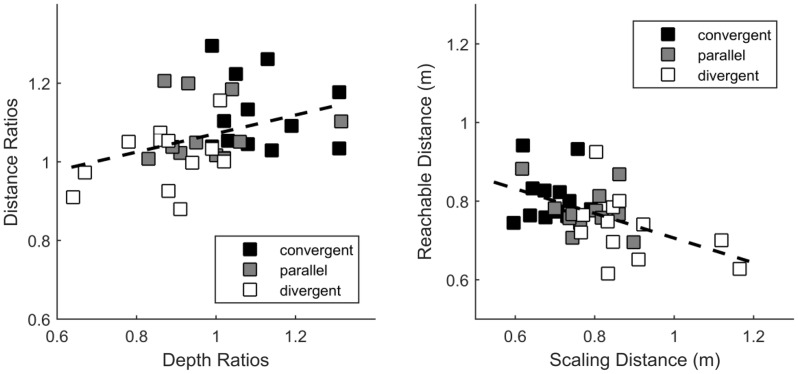
Scatter plots of depth ratios versus distance ratios (left) and of scaling distance versus reachable distance (right) for the three conditions of camera configuration (convergent, parallel, and divergent). Each dot corresponds to individual data. The dotted lines show the linear fits.

To further study how cameras configuration can affect distance and depth perception together, we compared data on perceived depth and data on perceived reachable distance (Figure 9). A correlational analysis was indeed justified by the fact that perceived depth from disparity depends on an estimate of distance. First, we analyzed the depth ratios observed in Experiment 4 and the distance ratios (perceived reachable distance over actual reachable distance) computed from Experiment 2. We performed the correlation over individual data so as to study how the depth-distance relationship fluctuates with changes in the cues to distance. We found a positive correlation between the depth ratios and the distance ratios (Pearson’s coefficient *R* = .38, *p* < .03, Figure 9, left). Then, we computed the scaling distances—the absolute distance at which the horizontal disparity pattern would provide the perception of a perfectly circular cylinder (Johnston, 1991)—based on depth estimations made in Experiment 4. We compared these scaling distances to the perceived reachable distances (actual reachable distance + judgment error) measured in Experiment 2. We observed a negative correlation between the scaling distance and the reachable distance (Pearson’s coefficient *R* = −.51, *p* < .002, Figure 9, right). These correlations were expected and consistent with the misperceptions caused by manipulating the horizontal pattern of vertical disparity.

## Discussion

We found that perceived depth is strongly affected by cameras configuration. Convergent cameras led observers to underestimate object depth (depth ratios > 1) whereas in the divergent cameras condition, observers overestimated object depth (depth ratios < 1).

One major objective of this study was to investigate how distance and depth perception are affected by convergent stereo-cameras. Correlation presented in Figure 9 (left) indicates that the underestimation of distance (i.e., the overestimation of reachable distance) is correlated with the underestimation of object depth. Furthermore, the effective distance used to scale binocular disparity in Experiment 4 increased as the perceived reachable distance decreased (Figure 7, right). These results show that distance perception and depth perception are affected in the same way by distance information, and therefore, they are consistent with the idea that the same distance estimate is used to perceive distance and to scale horizontal disparity (Brenner & van Damme, 1999).

This result, taken with the fact that the vertical disparity pattern is used to perceive the distance of surfaces (Rogers & Bradshaw, 1995), confirms that the information for distance perception is used for depth perception.

This conclusion is in agreement with the results of previous studies concerning the effect of vertical disparities on depth perception (e.g., Bradshaw et al., 1996; Garding, Porrill, Mayhew, & Frisby, 1995; Rogers & Bradshaw, 1993). The effect of vertical disparity on depth perception is indirect. Current models of cue combination assert that, because horizontal disparity is ambiguous, it must necessarily be scaled using an estimate of distance (Hillis et al., 2004; Landy et al., 1995). As this estimate of distance is influenced by the presence of vertical parallax, perceived depth can be indirectly affected. Such an indirect effect on perceived depth has also been demonstrated in studies of the effect of focus cues on absolute-distance perception in stereoscopic displays (e.g., Hoffman et al., 2008; Vienne, Blondé, & Mamassian, 2014).

## General Discussion

In this report, we have argued that manipulating vertical disparities by varying cameras configuration can affect distance and depth perception. We found that (a) the presence of a textured background surface reduces the uncertainty in judging the distance of a 3D object, (b) filming with convergent stereo-cameras introduces distortions that lead observers to underestimate the distance of objects in the scene as well as the divergent cameras condition leads observers to overestimate distance, (c) the effect of convergent cameras likely results from an unwanted vertical parallax and, that (d) the depth of objects is underestimated with convergent cameras and overestimated in the divergent cameras condition.

A former study (Allison, 2007) proposed that distortions in horizontal and vertical disparities produce perceptual effects in the opposite direction. Accordingly, a distortion in horizontal disparities (convex curvature) may lead viewers to perceive the more offset (peripheral) objects as being farther away. However, a distortion in vertical disparities suggests that the visual scene is nearer than it is. As a consequence, Allison (2007) suggested that these two types of distortions could counteract each together, cancelling their effects. However, different predictions can also be drawn depending on where the observer is looking at and on the structure of the scene. First, given the curvature effect, the perceived distance of an object lying on the sagittal plane is not the same as the one lying in the peripheral part of the image. Second, if several objects are present in the scene, relative disparities between them can be used to judge the distance of nearer objects (Sousa et al., 2010). For example, larger disparities between two objects lead observers to underestimate the distance of the nearer object (Sousa et al., 2010). As a consequence, distortions in horizontal disparities owing to convergent cameras might not necessarily lead to perceive objects as being farther away.

We have also found that the horizontal distortion arising from convergent cameras did not affect perceived distance. However, when the vertical and horizontal distortions were present, the perceived reachable distance was overestimated, suggesting that a distortion in vertical disparity is the main factor affecting perceived distance and depth.

A given pattern of horizontal disparity presented at a particular distance can give rise to the perception of a fronto-parallel surface (Rogers & Bradshaw, 2005). So, alternatively, one can suggest that observers might rely on a surface-planarity assumption to judge the distance of the surface. The pattern of horizontal disparity specifying a type of curvature could indicate the distance at which this pattern corresponds to a fronto-parallel surface. The hypothesis that observers assume surface-planarity is consistent with our report that the participants have barely seen a curved surface when vertical parallax was present (Experiments 2 and 4).

The manipulation of vertical disparity may also affect the perceived curvature by its indirect effect in scaling horizontal disparity. With convergent cameras, perceived distance is underestimated. Horizontal disparities should thus be scaled by a smaller apparent distance, potentially lessening the curvature effect. Rogers and Bradshaw (1995) found that to yield the perception of an apparent fronto-parallel surface, the surface needs to be more curved as the viewing distance decreased. As a result, the curvature might not be perceived because the vertical disparity introduced by cameras rotation has rescaled the horizontal disparity.

Another explanation for why observers did not perceive the curvature effect is related to the vertical parallax. The vertical parallax introduced by convergent cameras is also known to affect fusion ability and to cause visual discomfort (IJsselsteijn, de Ridder, & Vliegen, 2000). If the vertical disparity exceeds the vertical fusion zone, this can lessen the effect of horizontal disparities. Observers who cannot fuse stereo-images will not be able to use horizontal disparities as well. So, it can be suggested that the main effect of cameras configuration results from the distortion of vertical disparity.

We found that viewers misestimate the distance and depth of simulated objects displayed with convergent cameras, compared with parallel cameras. Viewers should be sensitive to these distortions in rich and structured environments, such as those often used in virtual–reality displays nowadays. These results lead us to support the use of parallel stereo-cameras or the use of a rectification processing to eliminate these distortions.

An interesting topic for future studies relates to the range of viewing distances over which the vertical disparity affects perceived depth or distance. Vertical disparities provide absolute-distance information (Rogers & Bradshaw, 1993), but these subtle differences between the retinas of both eyes also decrease with increasing viewing distance. Thus, the efficacy of vertical disparities could be reduced at far viewing distances. This range of distances is likely to be extended in camera configurations with increased inter-axial separation, resulting in hyper-stereo viewing (Newman & Ostler, 2009; Priot, Laboissiere, Sillan, Roumes, & Prablanc, 2010; Rogers, 2011).
